# Reinvestigation of Disulfide-bonded Oligomeric Forms of the Unfolded Protein Response Transducer ATF6

**DOI:** 10.1247/csf.19030

**Published:** 2019-12-19

**Authors:** Hibiki Koba, Shengyu Jin, Nanami Imada, Tokiro Ishikawa, Satoshi Ninagawa, Tetsuya Okada, Tetsushi Sakuma, Takashi Yamamoto, Kazutoshi Mori

**Affiliations:** 1 Department of Biophysics, Graduate School of Science, Kyoto University, Kyoto 606-8502, Japan; 2 Division of Integrated Sciences for Life, Graduate School of Integrated Sciences for Life, Hiroshima University, Hiroshima 739-8526, Japan

**Keywords:** disulfide-bonded structure, endoplasmic reticulum, membrane-bound transcription factor, non-reducing SDS-PAGE, unfolded protein response

## Abstract

ATF6α is an endoplasmic reticulum (ER)-embedded transcription factor which is rapidly activated by ER stress, and a major regulator of ER chaperone levels in vertebrates. We previously suggested that ATF6α occurs as a monomer, dimer and oligomer in the unstressed ER of Chinese hamster ovary cells due to the presence of two evolutionarily conserved cysteine residues in its luminal region (C467 and C618), and showed that ATF6α is reduced upon ER stress, such that only reduced monomer ATF6α is translocated to the Golgi apparatus for activation by proteolysis. However, mutagenesis analysis (C467A and C618A) revealed that the C618A mutant behaves in an unexpected manner (monomer and oligomer) during non-reducing SDS-PAGE, for reasons which remained unclear. Here, we used human colorectal carcinoma-derived HCT116 cells deficient in ATF6α and its relevant ATF6β, and found that ATF6α dimer and oligomer are both dimers, which we designated C618-dimer and C467-dimer, respectively. We demonstrated that C467-dimer (previously considered an oligomer) behaved bigger than C618-dimer (previously considered a dimer) during non-reducing SDS-PAGE, based on their disulfide-bonded structures. Furthermore, ATF6α monomer physically associates with another ATF6α monomer in the absence of disulfide bonding, which renders two C467 residues in close proximity so that formation of C467-dimer is much easier than that of C618-dimer. In contrast, C618-dimer is more easily reduced upon ER stress. Thus, our analysis revealed that all forms of ATF6α, namely monomer, C618-dimer and C467-dimer, are activated by single reduction of a disulfide bond in response to ER stress, ensuring the rapidity of ATF6α activation.

## Introduction

Protein must gain structure in three dimensions to function as assigned by genetic code. As protein misfolding constitutes a fundamental threat to all living cells, cells are equipped with various systems to counteract it (Bukau *et al.*, 2006). Protein unfolding or misfolding in the endoplasmic reticulum (ER), where newly synthesized secretory and transmembrane proteins are folded and assembled, activates the unfolded protein response (UPR) (Kaufman, 1999; Mori, 2000). As a result, translation is generally attenuated to decrease the burden on the folding machinery; transcription of ER-localized molecular chaperones is induced to augment folding capacity; and transcription of components of the ER-associated degradation machinery is induced to enhance degradation capacity. Together, these lead to maintenance of the homeostasis of the ER. These reactions are mediated by ubiquitously expressed transmembrane proteins in the ER, namely PERK, ATF6 and IRE1 in vertebrate cells, which somehow sense the accumulation of unfolded proteins in the ER and transmit signals to their respective downstream factors (Mori, 2009; Ron and Walter, 2007).

ATF6 does not exist in yeast; is present as a single gene product in non-vertebrates; and consists of two closely related forms in vertebrates, ATF6α and ATF6β, both of which are ubiquitously expressed. ATF6α and ATF6β are constitutively synthesized as type II transmembrane glycoproteins in the ER designated pATF6α(P) and pATF6β(P), respectively (Haze *et al.*, 2001, 1999). Upon ER stress pATF6α(P) and pATF6β(P) are relocated from the ER to the Golgi apparatus where they are subjected to sequential cleavage by Site-1 and Site-2 proteases (Okada *et al.*, 2003; Ye *et al.*, 2000). Their cytoplasmic regions containing the basic leucine zipper and transcriptional activation domains, designated pATF6α(N) and pATF6β(N), are released from the membrane and translocated into the nucleus where they induce the transcription of genes coding for quality control proteins in the ER, such as ER-localized molecular chaperones and components of ER-associated degradation (Adachi *et al.*, 2008; Yoshida *et al.*, 2000, 2001).

**Fig. 1:**
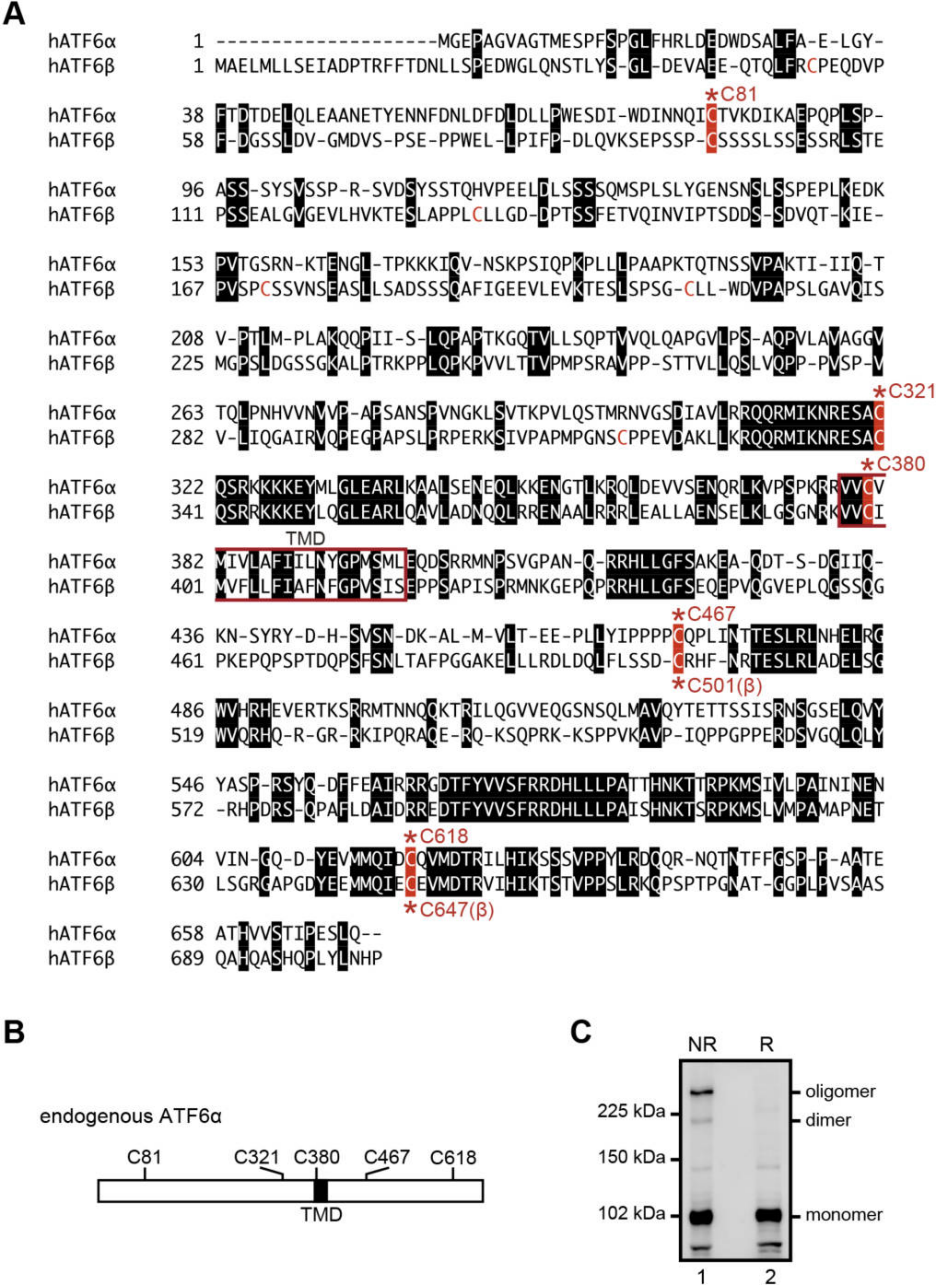
Amino acid alignment of human ATF6α and ATF6β. (A) ATF6α and ATF6β contain transmembrane domains (TMD, red square) in their middle. Amino acids identical between ATF6α and ATF6β are marked by white letters in black boxes. Five conserved cysteine residues are highlighted by white letters in red boxes with the asterisk and amino acid number. Two cysteine residues in the luminal region (C467 and C618 of ATF6α, and C501 and C647 of ATF6β) are highly conserved among vertebrates. (B) Schematic structure of ATF6α with the positions of TMD and five cysteine residues. (C) Immunoblotting using anti-ATF6α antibody of cell lysates prepared from WT HCT116 cells and then subjected to reducing (R) and non-reducing (NR) SDS-PAGE.

The luminal domains of both ATF6α and ATF6β have two conserved cysteine residues (C467 and C618 in human ATF6α; C501 and C647 in human ATF6β, see Fig. 1A and 1B). We previously suggested that they are present as monomer, dimer, and oligomer forms in unstressed ER of Chinese hamster ovary (CHO) cells but showed that they do not heterodimerize (Nadanaka *et al.*, 2007). Disulfide-bonded ATF6α was reduced upon treatment of cells not only with the reducing reagent dithiothreitol but also with the glycosylation inhibitor tunicamycin. Importantly, the extent of reduction correlated with that of activation, although reduction was not sufficient for activation; we did not observe a significant difference in kinetics for transport from the ER to the Golgi apparatus between wild-type (WT) and double cysteine mutant (C467&618A) of EGFP-ATF6α. Yet, only reduced ATF6α monomer reached the Golgi apparatus, and was a better substrate for Site-1 protease than the disulfide-bonded forms. We therefore proposed that this disulfide bonding-based mechanism ensures the strictness of regulation, in the sense that the cell can process ATF6α (and possibly also ATF6β) after it undergoes these changes in the ER.

In this paper, we aimed to answer the remaining question of why the C618A mutant of ATF6α exists as both a monomer and oligomer in unstressed ER, contrary to the C467A mutant which exists as expected as both a monomer and dimer, despite the presence of a single cysteine residue in their luminal regions. Because we assumed the presence of a putative protein X which mediates oligomerization of the C618A mutant (see Fig. 3D), we intended to confirm the presence of this protein X and then identify it. However, this line of investigation turned in an unexpected direction.

## Materials and Methods

### Construction of plasmids

Recombinant DNA techniques were performed according to standard procedures (Sambrook *et al.*, 1989) and the integrity of all constructed plasmids was confirmed by extensive sequencing analyses. pCGN-HA-ATF6α (Wang *et al.*, 2000) to express full-length ATF6α tagged with the hemagglutinin (HA) epitope at the N-terminus was the kind gift of R. Prywes (Columbia University, New York, NY). pCGN-HA-ATF6α(C467A), pCGN-HA-ATF6α(C618A), pCGN-HA-ATF6α(C467&618A), pCMVshort-EGFP-ATF6α(WT), pCMVshort-EGFP-ATF6α(C467A), pCMVshort-EGFP-ATF6α(C618A) and pCMVshort-EGFP-ATF6α(C467&618A) were constructed in previous studies (Nadanaka *et al.*, 2007; Nadanaka *et al.*, 2004). pCGN-HA-ATF6α(C81/321/380A) and pCGN-HA-ATF6α(C81/321/380/618A) were generated by inducing mutations in pCGN-HA-ATF6α and pCGN-HA-ATF6α(C618A), respectively, using DpnI-mediated site-directed mutagenesis. pcDNA-ssATF6α(C)WT-TAP and its cysteine mutants were constructed in a previous study (Sato *et al.*, 2011). pcDNA-ATF6α(WT)-TAP and its cysteine mutants were constructed by replacing ssATF6α(C) with full-length ATF6α(WT) or its cysteine mutants. The construction of pcDNA-ATF6α(Δ244)WT-TAP, pcDNA-ATF6α(Δ331)WT-TAP, pcDNA-ATF6α(Δ355)WT-TAP and their cysteine mutants were carried out by inverse PCR to remove the respective sequences from pcDNA-ATF6α(WT)-TAP or its cysteine mutants, followed by ligation using NEBuilder HiFi DNA Assembly Master Mix (New England Biolabs).

Transcription activator-like effector nuclease (TALEN) plasmids were constructed as described previously (Sakuma *et al.*, 2013). Each DNA-binding module was assembled into ptCMV-136/63-VR vectors using the two-step Golden Gate cloning method. Assembled sequences were 5'-TGAAATGGGGGAGCCGGCTggggttgccggcaCCATGGAGTCACCTTTTA-3' for ATF6α, and 5'-TGGCGGAGCTGATGCTGCtcagcgagattgctgACCCGACGCGTTTCTTCA-3' for ATF6β, where uppercase and lowercase letters indicate TALEN target sequences and spacer sequences, respectively.

### Cell culture and transfection

HCT116 cells (CCL-247; ATCC) were cultured in Dulbecco’s modified Eagle’s medium (DMEM; 4.5 g/l glucose) supplemented with 10% fetal bovine serum, 2 mM glutamine, and antibiotics (100 U/ml penicillin and 100 μg/ml streptomycin) at 37°C in a humidified 5% CO_2_/95% air atmosphere. Cells were transfected with plasmid DNA using Polyethylenimine MAX (Polysciences, Inc.) and then incubated at 37°C for an appropriate time to express the transfected gene.

### Construction of ATF6α targeting vector

The 1.2-kb fragment of the ATF6α gene used for the 3'-arm was amplified by PCR from HCT116 cell genomic DNA using the primers 5'-ATAAGAATGCGGCCGCTCGTGGTGACAGGTGTGGAC-3' and 5'-CCCAAGCTTACCTGGTCTGATCCTTCTTCTGC-3', and then inserted between the NotI and HindIII sites of the DT-A-pA/loxP/PGK-Puro-pA/loxP vector (Ninagawa *et al.*, 2014) to create the DT-A-pA/loxP/PGK-Puro-pA/loxP-3'-arm (ATF6α). The 0.6-kb fragment of the ATF6α gene used for the 5'-arm was amplified similarly using the primers 5'-GGGGTACCAGGGTAGACTCGCTTGGACTTTG-3' and 5'-CCGCTCGAGTGACGAGACGGTGGCCTAGT-3', and then inserted between the KpnI and XhoI sites of DT-A-pA/loxP/PGK-Puro-pA/loxP-3'-arm (ATF6α) to create pKO-ATF6α-Puromycin.

### Construction of ATF6β targeting vector

The 1.2-kb fragment of the ATF6β gene used for the 3'-arm was amplified by PCR from HCT116 cell genomic DNA using the primers 5'-ATAAGAATGCGGCCGCCACCGACAACCTGCTTAGCC-3' and 5'-ACGCGTCGACCCCACACCTCACACACCTCA-3', and then inserted between the NotI and SalI sites of the DT-A-pA/loxP/PGK-Neo-pA/loxP vector (provided by Laboratory for Animal Resources and Genetic Engineering, Center for Developmental Biology, Institute of Physical and Chemical Research, Kobe, Japan) to create the DT-A-pA/loxP/PGK-Neo-pA/loxP-3'-arm (ATF6β). The 0.6-kb fragment of the ATF6β gene used for the 5'-arm was amplified similarly using the primers 5'-GGGGTACCCCCTTGGGAACCTGGAAAAA-3' and 5'-CCGCTCGAGGCTCTACCGACCAGTAAGAGACCTG-3', and then inserted between the KpnI and XhoI sites of DT-A-pA/loxP/PGK-Neo-pA/loxP-3'-arm (ATF6β) to create pKO-ATF6β-Neomycin.

### Construction of ATF6α/β-DKO cells

pKO-ATF6α-Puromycin as well as TALEN 5' and 3' target vectors for ATF6α were transfected into HCT116 WT cells using Lipofectamine LTX (Invitrogen). Transfected cells were selected in DMEM containing puromycin (0.5 μg/ml). Puromycin-resistant colonies were isolated and the ATF6α-KO cell line was identified by immunoblotting and genomic PCR. Subsequently, pKO-ATF6β-Neomycin as well as TALEN 5' and 3' target vectors for ATF6β were transfected into HCT116 ATF6α-KO cells using Lipofectamine LTX. Transfected cells were selected in DMEM containing G418 (0.6 mg/ml). Neomycin-resistant colonies were isolated and the ATF6α/β-DKO cell line was identified by immunoblotting and genomic PCR.

### Genomic PCR

HCT116 cells were washed with ice-cold PBS, suspended in alkaline lysis reagent A (25 mM NaOH and 0.2 mM EDTA), boiled for 10 min and then mixed with an equivalent volume of alkaline lysis regent B (40 mM Tris/HCl, pH 8.0). Homologous recombination in HCT116 cells was confirmed by genomic PCR using a pair of primers: 5'-GGGGTACCCGCCCGGCCTTATTTTAGTTT-3' and 5'-AGCAACAGATGGAAGGCCTC-3' (primer set 1 for ATF6α); 5'-CCCGGTAGAATTAGCTTGGC-3' and 5'-GAGCCAAGATTGCGCCATTA-3' (primer set 2 for ATF6α); 5'-GGTGTCTAGTGGGATACAGAAGACTCG-3' and 5'-GCTACCAGGAAGTGTCCCACAGTTAT-3' (primer set 3 for ATF6α); 5'-CCGCTCGAGTGGCTTTGGGGTTTCCTTTC-3' and 5'-CCTCGATCGAGATCCGGAAC-3' (primer set 1 for ATF6β); 5'-CCCGGTAGAATTAGCTTGGC-3' and 5'-GCTAGATAACCAAAGGGGATGTGG-3' (primer set 2 for ATF6β); and 5'-CCGCTCGAGTGGCTTTGGGGTTTCCTTTC-3' and 5'-GCTAGATAACCAAAGGGGATGTGG-3' (primer set 3 for ATF6β).

### Immunoblotting

HCT116 cells were washed with ice-cold PBS and lysed in 300 μl of 1% NP-40 buffer (50 mM Tris/HCl, pH 7.5, containing 1% NP-40, 150 mM NaCl, protease inhibitor cocktail [Nacalai Tesque], 10 μM MG132 and 10 mM N-ethylmaleimide). Lysates were clarified by centrifugation at 14,000 rpm for 10 min at 4°C. Cleared lysates were mixed with 2× Laemmli’s SDS sample buffer with or without 200 mM dithiothreitol (reducing or non-reducing, respectively) and boiled for 5−10 min. Prepared cell lysates were subjected to SDS-PAGE followed by immunoblotting.

Immunoblotting was carried out according to the standard procedure (Sambrook *et al.*, 1989) using Western Blotting Luminol Reagent (Santa Cruz Biotechnology). Chemiluminescence was detected using an LAS-3000mini LuminoImage analyzer (Fuji Film). ATF6α and ATF6β were detected with rabbit anti-ATF6α (Haze *et al.*, 1999) and anti-ATF6β (Haze *et al.*, 2001) polyclonal antibodies, respectively. Mouse anti-β-actin monoclonal antibody was obtained from Wako Pure Chemical Industries. Mouse anti-Myc epitope monoclonal antibody conjugated with horseradish peroxidase was obtained from Medical and Biological Laboratories.

### Immunoprecipitation

HCT116 cells were lysed in 1% NP-40 buffer and clarified by centrifugation as described above. 200 μl of cleared lysates were incubated with 30 μl of 50% proteinG sepharose beads (GE Healthcare) for 90 min at 4°C with gentle shaking, and then centrifuged at 3,000 rpm for 1 min at 4°C to remove the sepharose beads. 200 μl of supernatants was incubated with 1 μl of mouse anti-HA epitope monoclonal antibody (Recenttec) or mouse anti-GFP monoclonal antibody (Roche Applied Science) overnight and then with 30 μl of 50% proteinG sepharose beads for 90 min at 4°C with gentle shaking. The sepharose beads were collected by centrifugation and washed with 1% NP-40 buffer twice and with PBS once. Immunoprecipitated materials were eluted by boiling for 10 min in 1× Laemmli’s SDS sample buffer containing 100 mM dithiothreitol, and then subjected to SDS-PAGE followed by immunoblotting.

## Results

### Molecular entity of ATF6α dimer and oligomer

**Fig. 2:**
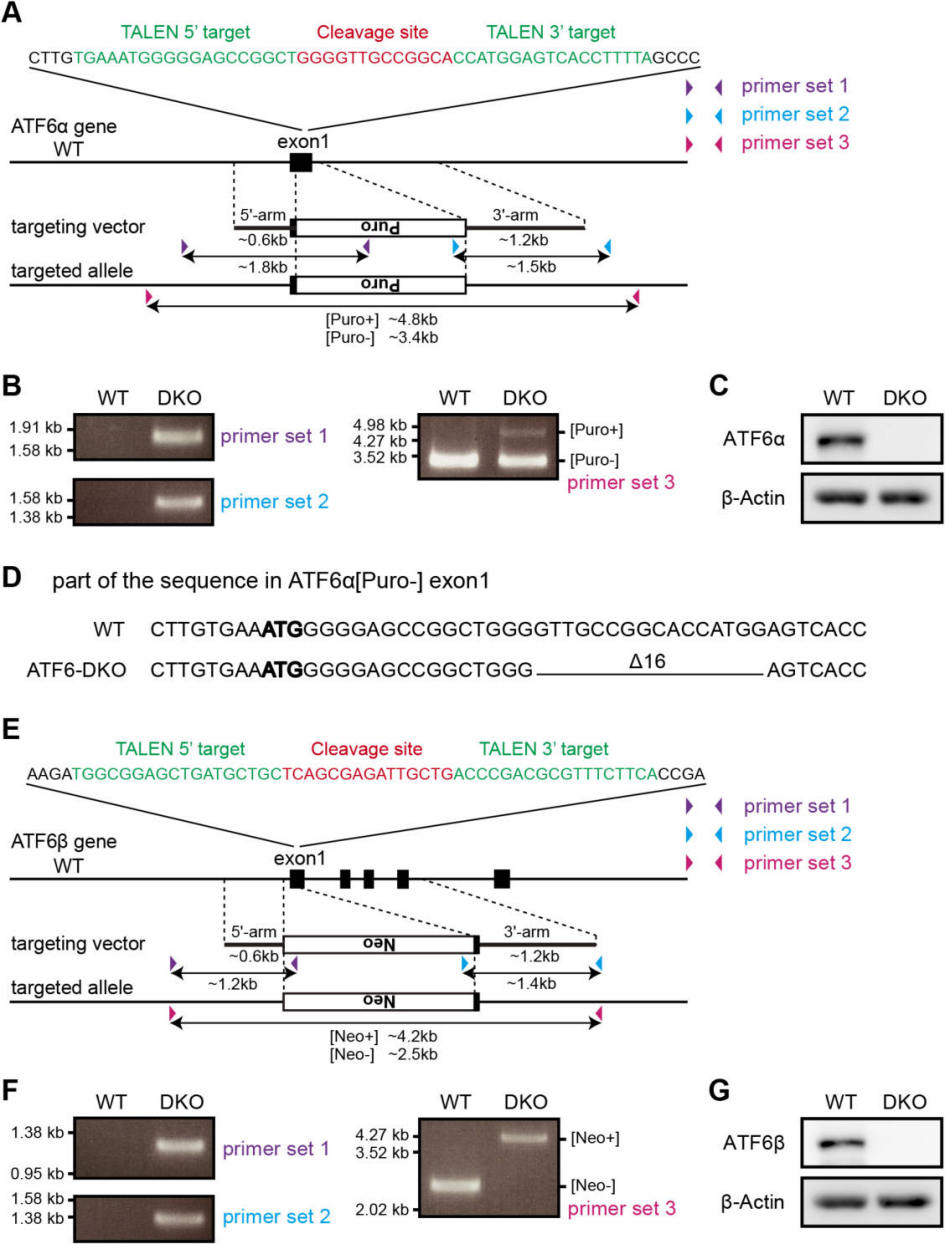
Establishment of ATF6α/β-DKO HCT116 cell line. (A) Strategy for ATF6α KO using the TALEN method. TALEN was designed to cleave exon 1 of ATF6α, and stimulate homologous recombination with the targeting vector. Colored triangles represent sites of primers for genomic PCR, and double-headed arrows represent regions to be amplified. (B) Genomic PCR to confirm homologous recombination in the ATF6α gene using the three primer sets shown in (A). (C) Immunoblotting using anti-ATF6α and β-actin antibodies of cell lysates prepared from HCT116 WT and DKO cells. (D) A part of the DNA sequence around the TALEN target site of ATF6α in the WT allele and DKO[Puro-] allele. In the DKO[Puro-] allele, 16 base pairs downstream of bold ATG, the start codon, are deleted, causing a frame shift. (E) Strategy for ATF6β KO using the TALEN method, described similarly to (A). (F) Genomic PCR to confirm homologous recombination in the ATF6β gene using the three primer sets shown in (E). (G) Immunoblotting using anti-ATF6β and β-actin antibodies of cell lysates prepared from HCT116 WT and DKO cells.

ATF6α occurs as a monomer, dimer and oligomer in unstressed ER of the HCT116 diploid cell line derived from human colorectal carcinoma (Roschke *et al.*, 2002), similarly to the case of CHO cells (Fig. 1C). To avoid interference from endogenous ATF6α and ATF6β in analyses of oligomer formation, we knocked out ATF6α in WT HCT116 cells and then knocked out ATF6β in ATF6α-knockout (KO) HCT116 cells to construct ATF6α/β-double KO (DKO) HCT116 cells by the TALEN method (Fig. 2A and 2E) (Joung and Sander, 2013). Expected homologous recombination was confirmed by genomic PCR (Fig. 2B and 2F), and a frame-shift-causing deletion in the ATF6α allele, into which the drug-resistant gene was not incorporated, was unraveled by DNA sequencing (Fig. 2D). The absence of ATF6α and ATF6β in ATF6α/β-DKO cells was confirmed by immunoblotting (Fig. 2C and 2G). We thus used ATF6α/β-DKO HCT116 cells (DKO cells hereafter) for subsequent analyses. Phenotypes of ATF6α-KO, ATF6β-KO, and ATF6α/β-DKO cells will be reported elsewhere.

**Fig. 3:**
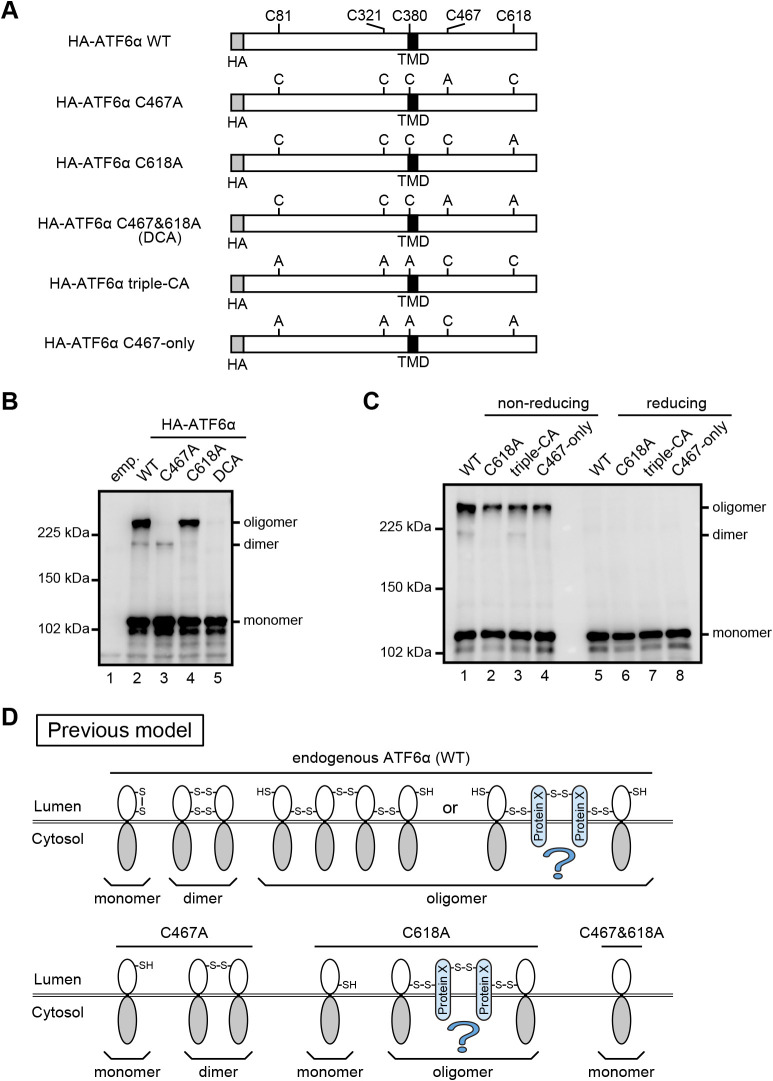
Oligomeric status of WT and various cysteine mutants of ATF6α. (A) Schematic structure of HA-tagged ATF6α WT with the positions of TMD and five cysteine residues (top) as well as those of its various cysteine to alanine mutants. (B) Immunoblotting using anti-ATF6α antibody of cell lysates prepared from HCT116 DKO cells expressing WT and cysteine mutants of HA-tagged ATF6α as indicated and then subjected to non-reducing SDS-PAGE. (C) Immunoblotting using anti-ATF6α antibody of cell lysates prepared from HCT116 DKO cells expressing WT and cysteine mutants of HA-tagged ATF6α as indicated and then subjected to reducing and non-reducing SDS-PAGE. (D) Previous model to explain the structures of dimer and oligomer produced from ATF6α WT, C467A, C618A, and C467&618A.

The HA-tagged C467A mutant of human ATF6α (Fig. 3A) occurred as a monomer and dimer (Fig. 3B, lane 3), whereas the HA-tagged C618A mutant (Fig. 3A) occurred as a monomer and oligomer (Fig. 3B, lane 4) in DKO cells, as previously shown in CHO cells (Nadanaka *et al.*, 2007). HA-tagged C467&618A mutant (DCA) (Fig. 3A) occurred only as a monomer, as expected (Fig. 3B, lane 5). We therefore postulated that the C618A mutant is disulfide-bonded to putative protein X to form an oligomer (Fig. 3D).

To exclude the possible involvement of non-luminal cysteine residues in oligomer formation and thereby confirm the presence of protein X, we simultaneously mutated three cysteine residues present in the cytoplasmic region (C81 and C321) and transmembrane domain (C380) of ATF6α (Fig. 3A). This HA-tagged triple-CA mutant behaved like HA-tagged WT (Fig. 3C, compare lanes 1 with 3), and the HA-tagged C618A mutant combined with the triple-CA mutation (C467-only, Fig. 3A) behaved like the HA-tagged C618A mutant (Fig. 3C, compare lanes 2 with 4) during non-reducing SDS-PAGE. These findings indicate that the C467-only mutant is still able to form an oligomer via disulfide bonding to protein X. Therefore, we tried to identify protein X by transfecting the HA-tagged C467-only mutant into HEK293 cells and by immuno-isolating the oligomer form. However, mass spectrometric analysis revealed that the obtained oligomer form was composed only of ATF6α (data not shown). These results prompted us to reinvestigate the molecular entity of ATF6 oligomer forms.

**Fig. 4:**
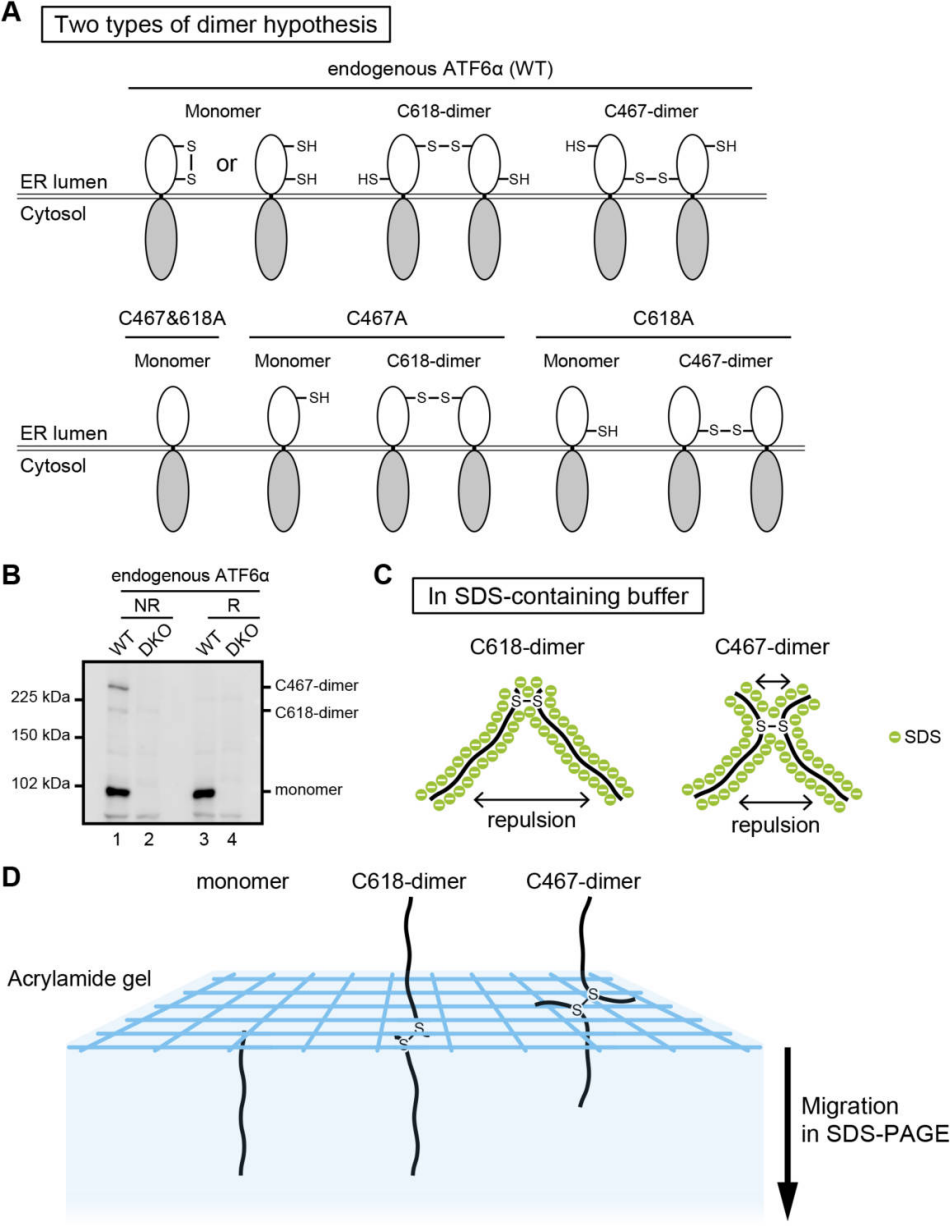
New model for ATF6α oligomer. (A) New model to explain the structures of dimer and oligomer produced from ATF6α WT, C467A, C618A, and C467&618A. (B) Immunoblotting using anti-ATF6α antibody of cell lysates prepared from WT and DKO HCT116 cells and then subjected to reducing (R) and non-reducing (NR) SDS-PAGE. (C) Schematic representation of C618-dimer and C467-dimer in SDS-containing buffer. (D) Model to explain the difference in migration speed between C618-dimer and C467-dimer.

**Fig. 5:**
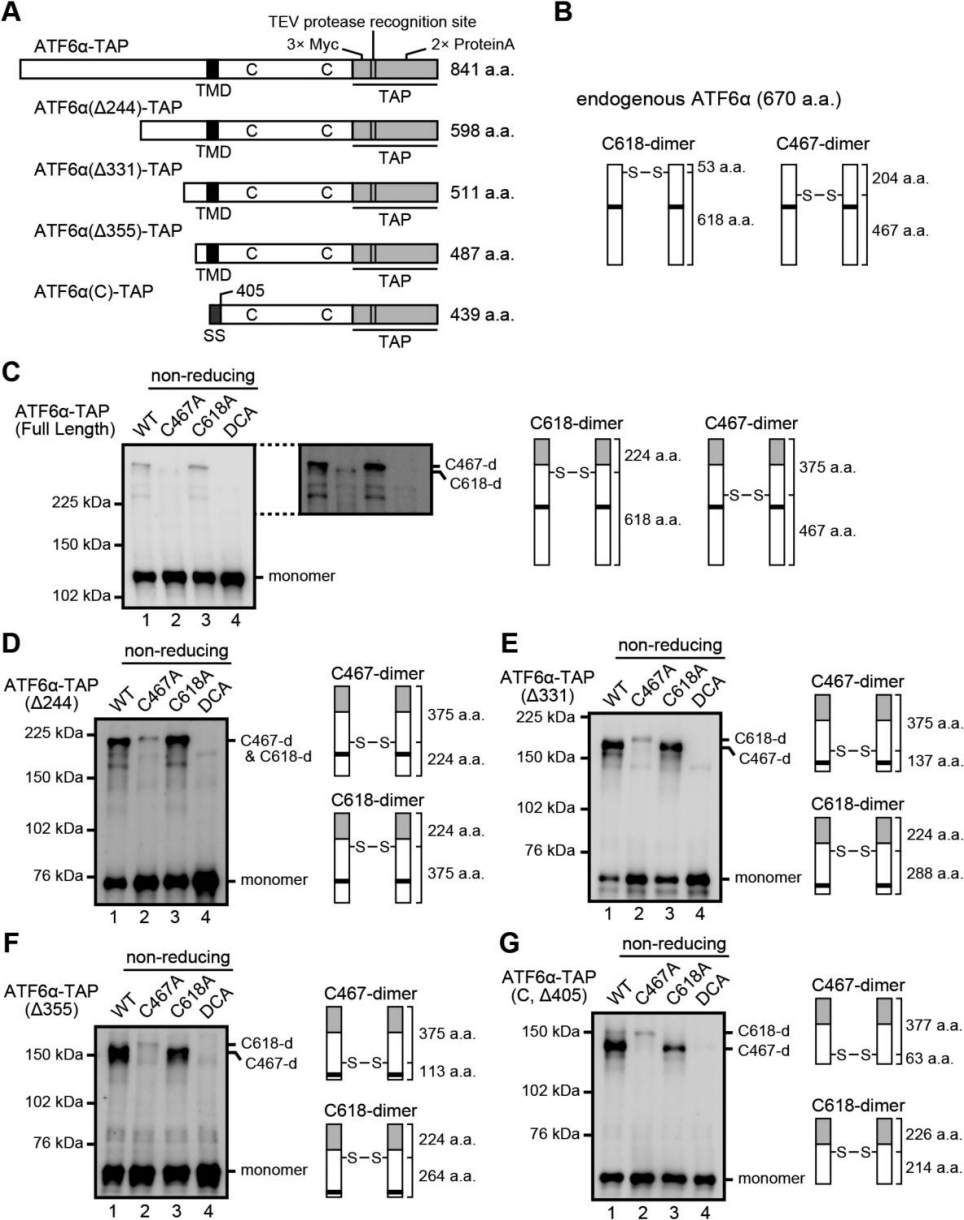
Migration speed of C618-dimer and C467-dimer of various lengths. (A) Schematic structures of ATF6α-TAP of various lengths at the N-terminus with the positions of TMD and two cysteine residues. SS denotes a signal sequence of mouse Immunoglobulin κ. (B) Schematic representation of C618-dimer and C467-dimer of endogenous ATF6α with the number of amino acid residues N-terminal and C-terminal to the respective disulfide bond. (C)–(G) Immunoblotting using anti-Myc antibody of cell lysates prepared from HCT116 DKO cells expressing WT and cysteine mutants of ATF6α-TAP with various lengths at the N-terminus and then subjected to non-reducing SDS-PAGE, as well as a schematic representation of their C618-dimer and C467-dimer with the number of amino acid residues N-terminal and C-terminal to the respective disulfide bond.

We hypothesized that the two higher molecular weight forms of ATF6α, which we presumed to be a dimer and an oligomer, are indeed both dimers (Fig. 4A), and that the dimer disulfide-bonded with C467 (referred to hereafter as C467-dimer) migrated more slowly than the dimer disulfide-bonded with C618 (referred to hereafter as C618-dimer) (Fig. 4B). Note that C467-dimer and C618-dimer are expected to have an X-like and upside-down V-like structures, respectively, in SDS-containing buffer (Fig. 4C). To understand the basis of why the two types of dimer behave differently during non-reducing SDS-PAGE, we added the tandem affinity purification (TAP) tag, which consists of 3x Myc, the TEV protease recognition site and 2x Immunoglobulin G-binding site of protein A to the C-terminus of ATF6α, and then introduced gradual deletions from the N-terminus to ATF6α-TAP to change the relative position of the disulfide bonds in the entire molecule (Fig. 5A). In addition, C467A, C618A, and C467&618A mutations were introduced into each of the ATF6α-TAP constructs (Fig. 5C–5G).

The C618-dimer produced from the C467A mutant of ATF6α-TAP migrated still faster than the C467-dimer produced from the C618A mutant of ATF6α-TAP, albeit only slightly, because the C618-dimer also became a more or less X-like structure after the addition of TAP (Fig. 5C). After introduction of a series of N-terminal deletions into ATF6α-TAP, the C618-dimer became more and more an X-like structure, whereas the C467-dimer became more and more a V-like structure. Accordingly, the C618-dimer with an X-like structure now migrated more slowly than the C467-dimer with a V-like structure, as we expected (Fig. 5D–5G). Based on these results we concluded that a protein with an X-like structure behaves bigger than a protein with a V-like structure during non-reducing SDS-PAGE (see *Discussion* for interpretation).

The conclusion that C467-dimer behaves bigger than C618-dimer based on their differential structures is likely to be applicable to C501-dimer (previously considered an oligomer) and C647-dimer (previously considered a dimer) of ATF6β, given that the positions of C501 and C647 of ATF6β are highly conserved with those of C467 and C618 of ATF6α (Fig. 1A).

### Disulfide bonding-independent dimerization of ATF6α monomer

**Fig. 6:**
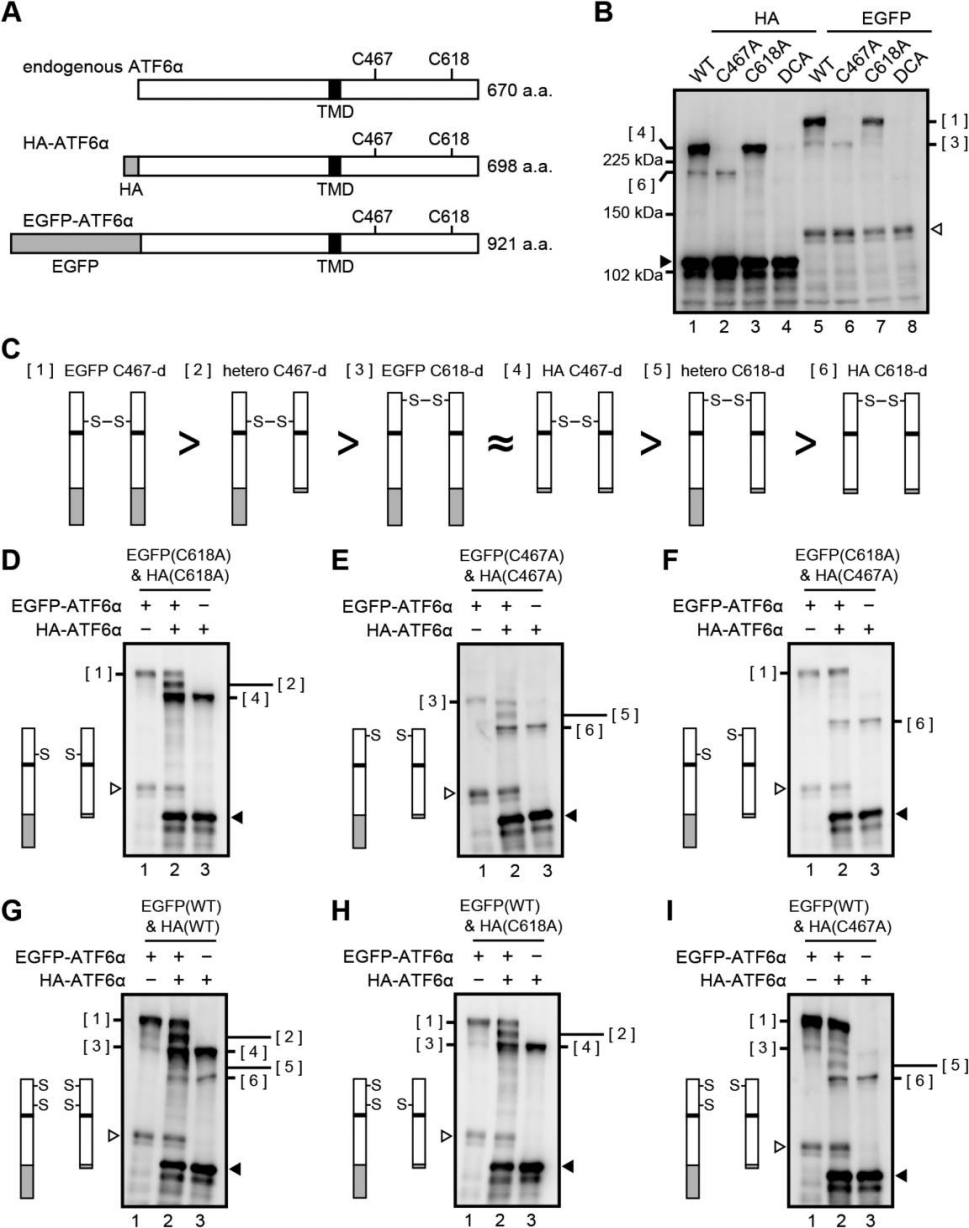
Visualization of ATF6α dimer formation. (A) Schematic structures of endogenous ATF6α, HA-ATF6α and EGFP-ATF6α with the positions of TMD and two cysteine residues. (B) Immunoblotting using anti-ATF6α antibody of cell lysates prepared from HCT116 DKO cells expressing WT and cysteine mutants of HA-ATF6α or EGFP-ATF6α and then subjected to non-reducing SDS-PAGE. Closed and open triangles denote monomer of HA-ATF6α and EGFP-ATF6α, respectively. (C) Schematic representation of various disulfide-bonded dimers. Each greater-than symbol implies that a dimer on the left behaves bigger than that on the right during non-reducing SDS-PAGE. The approximation symbol implies that dimers [3] and [4] migrate to similar positions during non-reducing SDS-PAGE. (D)–(I) Immunoblotting using anti-ATF6α antibody of cell lysates prepared from HCT116 DKO cells expressing various combinations of WT and cysteine mutant of HA-ATF6α and EGFP-ATF6α as indicated by the schematic structure on the left and then subjected to non-reducing SDS-PAGE. Molecular entity of the faint band around the position of [3], which is observed when only HA-ATF6α(C467A) was expressed by transfection (E, lane 3; F, lane 3; I, lane 3), remains unclear.

To strengthen our hypothesis, we devised a way to observe the dimerization of ATF6α more directly. To this end, we constructed WT and mutant versions (C467A, C618A, C467&618A=DCA) of ATF6α tagged with EGFP at the N-terminus (Fig. 6A). C467-dimer (see Fig. 6C[1]) and C618-dimer (see Fig. 6C[3]) of EGFP-ATF6α migrated more slowly than C467-dimer (see Fig. 6C[4]) and C618-dimer (see Fig. 6C[6]) of HA-ATF6α, respectively (Fig. 6B), confirming that migration speed during non-reducing SDS-PAGE correlates with the length of the ATF6α molecule. Co-expression of EGFP-ATF6α(C618A) and HA-ATF6α(C618A) (Fig. 6D) or co-expression of EGFP-ATF6α(C467A) and HA-ATF6α(C467A) (Fig. 6E) unequivocally produced a band of heterodimer (Fig. 6C[2] or Fig. 6C[5]), whereas co-expression of EGFP-ATF6α(C618A) and HA-ATF6α(C467A) did not produce a heterodimer band (Fig. 6F), indicating that ATF6 is dimerized only through disulfide bonding between two C467 residues or between two C618 residues. This notion was confirmed by co-expression of EGFP-ATF6α(WT) and HA-ATF6α(WT) (Fig. 6G), EGFP-ATF6α(WT) and HA-ATF6α(C618A) (Fig. 6H), and EGFP-ATF6α(WT) and HA-ATF6α(C467A) (Fig. 6I).

**Fig. 7:**
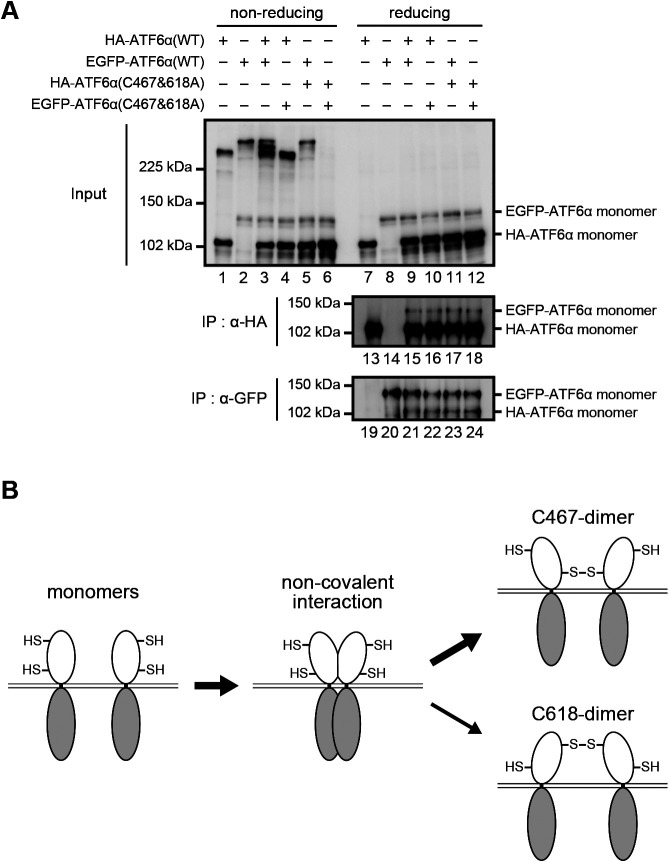
Non-covalent association of ATF6α monomers. (A) Immunoblotting using anti-ATF6α antibody of cell lysates prepared from HCT116 DKO cells expressing one or two of HA-ATF6α(WT), EGFP-ATF6α(WT), HA-ATF6α(C467&618A), and EGFP-ATF6α(C467&618A) as indicated and then subjected to reducing and non-reducing SDS-PAGE, as well as of immunoprecipitates obtained using anti-HA or anti-GFP antibodies from cell lysates as indicated and then subjected to reducing SDS-PAGE. (B) Model for the preferential formation of C467-dimer to that of C618-dimer.

To gain further insight into the dimerization of ATF6α, we asked whether ATF6α monomer associates with ATF6α monomer without disulfide bonding. To this end, WT and the C467&618A mutant of HA-ATF6α and EGFP-ATF6α were transfected into DKO cells and immunoprecipitation was carried out using anti-HA and GFP antibodies under non-denaturing (disulfide bond-retaining) conditions. Immunoblotting of cell lysates after non-reducing and reducing SDS-PAGE showed the expected bands: HA-ATF6α(WT) heterodimerized with EGFP-ATF6α(WT) via disulfide bonding (Fig. 7A, lane 3) but not with EGFP-ATF6α(C467&618A) (Fig. 7A, lane 4); EGFP-ATF6α(WT) did not heterodimerize with HA-ATF6α(C467&618A) (Fig. 7A, lane 5); and HA-ATF6α(C467&618A) did not heterodimerize with EGFP-ATF6α(C467&618A) (Fig. 7A, lane 6). Importantly, immunoblotting of immunoprecipitates after reducing SDS-PAGE showed that similar amounts of EGFP-ATF6α were immunoprecipitated with anti-HA antibody (Fig. 7A, lanes 15–18) and similar amounts of HA-ATF6α were immunoprecipitated with anti-GFP antibody (Fig. 7A, lanes 21–24) from DKO cells expressing HA-ATF6α and EGFP-ATF6α, regardless of WT or C467&618A. Thus, ATF6α monomer tends to form dimer non-covalently even in the absence of disulfide bonding, although we could not quantify the extent of this non-covalent association over disulfide-bonded association.

## Discussion

We previously suggested that ATF6α occurs in the ER of CHO cells as oxidized monomer, dimer and oligomer, based on their migration positions during non-reducing SDS-PAGE. Our pulse-labeling experiment showed that the monomer is formed first, followed by formation of the dimer and oligomer. Our pulse-chase experiment showed that the oligomer is not a dead-end product, as all forms are decreased after chase (Nadanaka *et al.*, 2007). Here, we showed that ATF6α exists in the ER of HCT116 cells as monomer, C618-dimer and C467-dimer, but not as oligomer. Considering that we did not observe a significant difference in migration position of the monomer between non-reducing and reducing SDS-PAGE (Fig. 1C), in marked contrast to the case of CHO cells (Nadanaka *et al.*, 2007), ATF6α monomer might not be oxidized in HCT116 cells (see Fig. 4A). It should be noted that a reduction in ATF6α is not sufficient for activation of ATF6α (Nadanaka *et al.*, 2007).

Repulsion between negative charges of SDS attached to polypeptide chains occurs with both C618-dimer and C467-dimer after the addition of SDS to cell lysates and boiling (Fig. 4C). As a result, C618-dimer with a V-like structure becomes an almost linear polypeptide via the free rotation of disulfide bonding (Fig. 4D) and migrates to a position expected from its molecular weight of ~200 kDa during non-reducing SDS-PAGE (Fig. 4B). In contrast, C467-dimer, with an X-like structure, still contains two short overhangs, which we believe explains why C467-dimer behaves bigger than C618-dimer during non-reducing SDS-PAGE (Fig. 4B and 4D).

In both CHO cells and HCT116 cells, C467-dimer is far more abundant than C618-dimer (Fig. 4B), although C618-dimer is more stable than C467-dimer in CHO cells (Nadanaka *et al.*, 2007), suggesting that stability is not always a basis for abundance. In this connection, we found that ATF6α monomer can physically associate with another ATF6α monomer even without disulfide bonding (Fig. 7A). This association might result in two C467 residues occurring in close proximity, such that the formation of C467-dimer would be much easier than that of C618-dimer (Fig. 7B). Instead, C618-dimer would be more easily reduced in response to ER stress. It should be noted that C618-dimer (previously called dimer) initially decreased in CHO cells treated with dithiothreitol or tunicamycin (Nadanaka *et al.*, 2007).

Because the reduction of ATF6α is an important step in its activation in response to ER stress, reducing enzymes for ATF6α have been sought. Two candidates have been identified, namely PDIA5 (Higa *et al.*, 2014) and ERp18 (Oka *et al.*, 2019), although the entire mechanism of the reduction remains unclear. During this search, Oka *et al.* also carried out mass spectrometric analysis of C467-dimer of ATF6α(C618A) (previously presumed protein X-containing oligomer) and found that it contained only ATF6α, consistent with our present analysis and new model.

In conclusion, our results reveal that all forms of ATF6α, namely the monomer, C618-dimer and C467-dimer, are activated by single reduction of disulfide bond in response to ER stress for subsequent transport to the Golgi apparatus via COPII vesicles. Among ubiquitously expressed UPR sensors, IRE1 is conserved from yeast to human; PERK is gained in metazoan; and ATF6 becomes functional in vertebrates. It is well known that the activation of XBP1, a downstream transcription factor of IRE1, is slower than that of ATF6, as XBP1 mRNA must be first spliced and then translated to produce the active form of XBP1 (Mori, 2003). This rapid activation of ATF6α might have been a key characteristic when the major regulator of ER chaperone levels switched from IRE1 to ATF6α during evolution, perhaps with the advent of vertebra (Ishikawa *et al.*, 2013; Mori, 2009).

## Abbreviations

CHO: Chinese hamster ovary
DKO: double knockout
ER: endoplasmic reticulum
HA: hemagglutinin
KO: knockout
TALEN: transcription activator-like effector nuclease
TAP: tandem affinity purification
UPR: unfolded protein response
WT: wild-type
